# Online Transdiagnostic Emotion Regulation Treatment for Adolescents With Mental Health Problems: A Randomized Clinical Trial

**DOI:** 10.1001/jamanetworkopen.2025.14871

**Published:** 2025-06-11

**Authors:** Katja Sjöblom, Katri Frankenstein, Lars Klintwall, Jannike Nilbrink, Maria Zetterqvist, Hugo Hesser, Erik Hedman-Lagerlöf, James J. Gross, Clara Hellner, Martin Bellander, Johan Bjureberg

**Affiliations:** 1 Department of Clinical Neuroscience, Centre for Psychiatry Research, Karolinska Institutet, and Stockholm Health Care Services, Region Stockholm, Stockholm, Sweden; 2 Department of Child and Adolescent Psychiatry in Linköping, Region Östergötland, and Center for Social and Affective Neuroscience, Department of Biomedical and Clinical Sciences, Linköping University, Linköping, Sweden; 3 School of Behavioural, Social and Legal Sciences, Örebro University, Örebro, Sweden; 4 Department of Clinical Neuroscience, Division of Psychology, Karolinska Institutet, Stockholm, Sweden; 5 Department of Psychology, Stanford University, Stanford, California

## Abstract

**Importance:**

Mental health problems are common during adolescence, but access to effective treatments is limited. Transdiagnostic treatments could address this treatment gap, but their feasibility, acceptability, and effectiveness remain unknown.

**Objective:**

To test the feasibility and acceptability of an online emotion regulation treatment for adolescents with mental health problems and investigate the preliminary effects on clinical outcomes and the target mechanism, emotion regulation.

**Design, Setting, and Participants:**

This single-blind randomized clinical trial was conducted between October 16, 2022, and July 28, 2023, in a primary care setting in Sweden. Participants in the intention-to-treat analysis were adolescents aged 12 to 17 years with mental health problems and their parents.

**Intervention:**

Participants were randomized 1:1 to 6 weeks of therapist-guided online transdiagnostic emotion regulation treatment or an active control condition consisting of 6 weeks of online supportive treatment.

**Main Outcomes and Measures:**

The primary outcomes were feasibility and acceptability measures, including consent rate, completion of assessments, adherence, credibility and expectancy ratings (Credibility/Expectancy Questionnaire), and treatment satisfaction (Client Satisfaction Questionnaire), immediately after treatment. Clinical outcomes, rated by blinded assessor, included global symptom severity and improvement, symptoms of depression and anxiety, global functioning, and emotion regulation.

**Results:**

A total of 30 adolescents (mean [SD] age, 14.2 [1.48] years; 28 females [93%]) were randomized to experimental treatment (n = 15) or active control treatment (n = 15). The consent rate (30 of 37 eligible participants [81%]) and rate of assessment completion immediately after treatment (28 [93%]) were high. Adherence, credibility, expectancy, and satisfaction in both groups were adequate. Participation in the experimental condition, but not the control condition, was associated with large within-group reductions in symptom severity (effect size, 1.30; 95% CI, 0.73-1.86) and symptoms of anxiety and depression (Cohen *d*, 1.07; 95% CI, 0.37-1.84), improved global functioning (Cohen *d*, 1.26; 95% CI, 0.66-1.85), and reductions in maladaptive cognitive coping (Cohen *d*, 1.10; 95% CI, 0.52-1.70) immediately after treatment.

**Conclusion and Relevance:**

In this randomized clinical trial, a brief online transdiagnostic emotion regulation treatment targeting adolescents with mental health problems was found to be feasible, acceptable, and potentially efficacious in primary care and may increase treatment outreach and accessibility for this population.

**Trial Registration:**

ClinicalTrials.gov Identifier: NCT05032547

## Key Points

**Question:**

Is a brief online transdiagnostic emotion regulation treatment for adolescents with mental health problems feasible, acceptable, and potentially efficacious?

**Findings:**

In this single-blind randomized clinical trial including 30 adolescents and their parents, a brief online transdiagnostic emotion regulation treatment was deemed feasible and acceptable. Large significant improvements in mental health symptoms and reductions in maladaptive emotion regulation strategies were found for the experimental group but not for the active control condition.

**Meaning:**

These findings suggest that a brief online transdiagnostic emotion regulation treatment holds promise for addressing mental health problems in adolescents in primary care settings.

## Teaser

This randomized clinical trial examines the feasibility, acceptability, and efficacy of a brief online transdiagnostic emotion regulation treatment for adolescents with mental health problems.

## Introduction

Mental health problems and mental disorders affect young people worldwide.^1,2^ Comorbidity between different mental health disorders is prevalent,^3^ and subthreshold disorders significantly contribute to psychiatric morbidity, representing a major burden.^4^ There is a shortage of treatment for mental health problems,^5^ and structural barriers, such as limited availability of professional services and excessive waiting times, hinder adolescents from receiving treatment.^6^ Online transdiagnostic psychological treatments have the potential to address these barriers^7^ and hold particular promise in treating comorbid, subthreshold, and suprathreshold problems.^8^

Existing psychological treatments for adolescents often focus on a subset of mental disorders, do not address subthreshold or multidisorder mental health problems, and have varying levels of efficacy.^9^ Maladaptive emotion regulation strategies are related to a wide range of mental health problems, suggesting that emotion regulation can be an underlying target mechanism in psychological interventions.^10^ Interventions targeting mental health problems and emotion regulation in adolescents have shown potential to improve emotion regulation abilities and mental health problems.^11,12,13^ To this end, we developed a brief, online, transdiagnostic, therapist-guided emotion regulation treatment called Primary Care Online Emotion-Regulation Treatment (POET).

The first aim of the current trial was to investigate the feasibility and acceptability of POET compared with an active control treatment. Secondary aims were to investigate the effect on clinical outcomes and the target mechanism.

## Methods

### Design

This single-blind randomized clinical trial compared POET with an active control treatment (hereafter, supportive treatment) for adolescents with mental health problems (NCT05032547). One parent of each adolescent completed a parallel 6-week online course. The trial was conducted in a primary care setting in Sweden. Recruitment took place from October 16, 2022, to January 31, 2023. The primary end points were measures immediately after treatment. Secondary end points were measures 3 months after treatment (ended July 28, 2023). We followed the Consolidated Standards of Reporting Trials (CONSORT) reporting guideline for randomized clinical trials. The trial was approved by the Swedish Ethical Review Authority. All participants provided informed consent. Participants aged 15 years or older and parents provided written consent. Younger participants provided verbal consent with parental written consent. The trial protocol is in Supplement 1.

### Participants

Self-referrals and referrals from health care professionals were accepted. The trial was advertised in primary care settings and on social media. Inclusion criteria were (1) age of 12 to 17 years (individuals younger than 18 years could participate), (2) presence of mental health problems, and (3) having at least 1 parent willing to participate in the parent course. Exclusion criteria included (1) severe mental illness requiring specialized care or low global functioning corresponding to a Children’s Global Assessment Scale (CGAS)^14^ score of less than 41 on a scale of 1 to 100 (higher scores indicating better functioning), (2) acute suicidality, (3) ongoing psychological treatment, (4) changes in psychopharmacological medication during the past 2 months, (5) insufficient skills in speaking and understanding Swedish, and (6) life circumstances that could prevent treatment participation.

### Procedures

The process for participating in the trial involved an initial telephone screening, collection of informed consent, and baseline assessments. Potential participants and parents completed an assessment interview over video link, including a semistructured interview^15^ administered by a licensed psychologist (K.S., K.F., L.K.) to assess eligibility. Eligible participants were randomly allocated (1:1) to either POET or supportive treatment. Randomization was conducted by an independent researcher using a true random number service (generating randomness via atmospheric noise). The random allocation sequence was placed in opaque sealed envelopes. Outcome measures comprised both blinded assessor–rated telephone interviews and online self-reported assessments immediately after and 3 months after treatment. Assessors were blinded to treatment allocation, and blinding integrity was assessed by asking assessors to guess allocation and report any disclosed group allocation.

### Outcomes

#### Feasibility and Acceptability

The feasibility outcomes were (1) consent rate, defined as the proportion of eligible participants who consented, and (2) completion of assessments (completing at least 1 clinical outcome immediately after treatment). Acceptability outcomes were (1) adherence to treatments (completing at least 3 modules); (2) youth ratings of credibility and expectancy of treatment, measured with the Credibility/Expectancy Questionnaire (CEQ; credibility score range, 1-9; expectancy score range, 0%-100%, with higher scores indicating greater credibility and expectancy)^16^ after completing the first module; and (3) youth and parent satisfaction with treatment or course, measured with the Client Satisfaction Questionnaire (CSQ-8; score range, 8-32, with higher scores indicating greater satisfaction)^17^ administered immediately after treatment. Therapist time and the feasibility of the blinding procedure were monitored. Adverse events were recorded by therapists, and participants were asked to report any potential adverse events and their impact during treatment.

#### Clinical Outcomes

Clinical outcomes were (1) symptom severity and improvement, measured with the Clinical Global Impressions–Severity Scale (CGI-S) and CGI–Improvement Scale (CGI-I)^18^; (2) symptoms of anxiety and depression, assessed with the Revised Child Anxiety and Depression Scale (RCADS-47)^19^; and (3) global functioning, measured with the CGAS.^14^ Before randomization, clinical outcomes (except for CGI-I) were measured by a clinician (K.S., K.F., L.K.), and outcomes at both time points were measured by blinded assessors. Treatment response was defined as a CGI-I rating of 1 (very much improved) or 2 (much improved).^20,21^

### Target Mechanism

Facets of emotion regulation, the proposed target mechanism, were measured with (1) the Cognitive Emotion Regulation Questionnaire (CERQ),^22^ measuring adaptive and maladaptive strategies, and (2) the Perth Alexithymia Questionnaire–Short Form,^23^ measuring alexithymia. Measures assessing the target mechanism were rated by adolescents before treatment, immediately after treatment, and 3 months after treatment. Information on all measures is provided in the eAppendix in Supplement 2.

### Interventions

The theoretical foundation of POET is the Extended Process Model of Emotion Regulation.^24^ This model describes the emotion-generative sequence and 4 different families of strategies used to regulate emotions: situational, attentional, cognitive, and response modulation. POET builds on the internet-delivered Emotion Regulation Individual Therapy for Adolescents (IERITA), targeting youths with nonsuicidal self-injury; IERITA was developed and evaluated by the current research group.^25,26^ POET aims to improve adolescents’ emotion regulation abilities through 6 modules. The modules focus on psychoeducation on emotions and skill training in emotion regulation strategies aligned with the Extended Process Model of Emotion Regulation.^24^ Parents enrolled in an online course providing skill training in supporting the adolescent in using more adaptive and fewer maladaptive emotion regulation strategies.

Due to the challenges in defining routine care in Swedish primary care and the absence of standardized treatment recommendations and significant disparities in access to interventions, an active control condition was chosen.^27,28^ This approach maximizes control over nonspecific effects, minimizes expectation biases, and enhances internal validity. The control treatment mimicked supportive therapy, a well-established comparator in adolescent clinical trials.^29,30^ It included no active elements of POET; rather, it consisted of information on mental health, and participants were encouraged to reflect on themes related to well-being (eg, school and friends).^29^ Parents enrolled in an online course including weekly reflections on how to support their adolescent’s well-being.

Both treatments were delivered across 6 weeks in a blended treatment format combining 6 online modules for adolescents and parents with therapist-guided sessions delivered over video link. Both treatments included therapist support, including feedback on modules and homework. Therapists delivering the treatments (K.S., K.F., L.K.) were clinical psychologists working in primary care in Sweden. A description of the interventions can be found in eTables 1 to 4 and eFigures 1 to 3 in Supplement 2.

### Statistical Analysis

Following the CONSORT 2010 Statement extension for feasibility randomized clinical trials, a sample size of 30 participants was considered appropriate for assessing feasibility and acceptability in this trial.^31^ In addition, an a priori power calculation indicated 30 participants would provide 80% power to detect a meaningful standardized mean difference of 0.6 between pretreatment and posttreatment measurement within each condition given an α level of 0.05 and accounting for 20% dropout.

Feasibility and acceptability outcomes are presented using descriptive statistics. Differences between treatments in satisfaction and therapist time were analyzed using independent sample *t* tests. To test whether blinded assessors’ guesses on treatment allocation differed from chance, a binomial test was used. This feasibility trial was not powered to robustly detect group differences for the clinical outcome and target mechanism. However, within-group effects were evaluated in an exploratory manner according to the intention-to-treat principle. The data were visually inspected using histograms to check for distribution assumptions and outliers.

Linear quantile mixed models for ordinal outcomes and linear mixed-effects regressions for continuous outcomes were fitted for the POET and supportive treatment groups separately. These included a random intercept for participant and a dummy-coded time variable (before treatment, after treatment, and 3 months after treatment, with the pretreatment time point as the reference category).

Because the CGI-S outcome is ordinal, median effects were estimated with a linear quantile mixed model using the lqmm package in R, version 4.4.1 (R Project for Statistical Computing).^32^ *P* values and 95% CIs were calculated using the bootstrap procedure implemented in the lqmm package, with 1000 replications. The effect size was calculated by dividing the model estimates of the median effect of time by the SD of the outcome at the first time point (this is analogous to the Cohen *d* for mixed models). For CGI-I, the number of participants classified as responders is presented.

Mixed-effects regression analyses for repeated measures (including all assessment points) provide unbiased estimates (and SEs) under the assumption that the data are missing at random. This assumes that given the observed data, the probability of missingness depends only on observed variables and not on unobserved data.^33^ Effect sizes were evaluated with Cohen *d* for mixed-effects models by dividing the unstandardized β coefficient for the time variable by the SD at the first time point, with 95% bootstrap (1000 simulations) CIs.^34^ Signs of effect sizes were changed so that a positive sign indicates improvement between time points. All tests were 2-sided, statistical significance was set at *P* < .05, and R, version 4.4.1 was used for statistical analysis.

## Results

**Figure 1.**
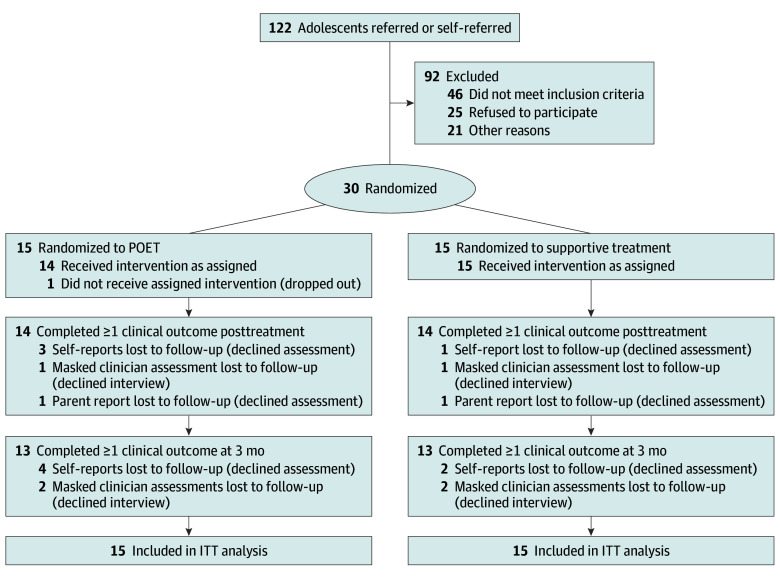
Flow Diagram of Patient Enrollment and Disposition Participants without follow-up data were included based on their baseline values following the intention-to-treat (ITT) approach. POET indicates Primary Care Online Emotion-Regulation Treatment.

In this study, 30 participants (28 [93%] female, 2 [7%] male) aged 12.1 to 17.5 years (mean [SD] age, 14.2 [1.48] years) were recruited and randomly allocated to POET (n = 15) or supportive treatment (n = 15). Figure 1 depicts the participant recruitment flowchart, and Table 1 shows participant characteristics. Twenty-four participants (80%) had previous contact with health care due to mental health problems. Eleven participants (37%) were referred by primary care or by a school health team. Nineteen participants (63%) were self-referred, with 15 (50%) referring themselves after responding to a social media advertisement. One participant (3%) in the POET arm dropped out before initiating the treatment due to lack of motivation.

**Table 1.** Study and Participant Characteristics

Characteristic	Participants, No. (%)		
	POET (n = 15)	Supportive treatment (n = 15)	Total (N = 30)
Sex			
Female	15 (100)	13 (86)	28 (93)
Male	0	2 (13)	2 (7)
Age, mean (SD), y	14.2 (1.58)	14.2 (1.43)	14.2 (1.48)
Parental role			
Mother	13 (87)	11 (73)	24 (80)
Father	2 (13)	3 (20)	5 (17)
Other	0	1 (7)	1 (3)
Parental educational level			
Secondary school	1 (7)	1 (7)	2 (7)
College or university <3 y	5 (33)	0	5 (17)
College or university ≥3 y	9 (60)	14 (93)	23 (77)
Parent employed or self-employed	15 (100)	15 (100)	30 (100)
Mental disorders^a^			
Major depressive disorder	5 (33)	2 (13)	7 (23)
Anxiety disorders			
Panic disorder	1 (7)	1 (7)	2 (7)
Separation anxiety disorder	0	1 (7)	1 (3)
Specific phobia disorder	3 (20)	2 (13)	5 (17)
Social anxiety disorder	6 (40)	4 (27)	10 (33)
Generalized anxiety disorder	1 (7)	1 (7)	2 (7)
ADHD^b^	2 (13)	1 (7)	3 (10)
Previous contact with health care due to mental health problems	14 (93)	9 (60)	24 (80)
Ongoing psychopharmacological medication (ATC code)^c^			
Hypnotics			
Melatonin (N05CH01)	1 (7)	1 (7)	2 (7)
Antihistamines (R06A)	0	1 (7)	1 (3)
Psychostimulants			
Lisdexamfetamine (N06BA12)	1 (7)	1 (7)	2 (7)
Methylphenidate (N06BA04)	1 (7)	0	1 (3)
Antidepressants			
SSRI (N06AB)	0	1 (7)	1 (3)

Abbreviations: ADHD, attention-deficit/hyperactivity disorder; ATC, Anatomical Therapeutic Chemical; POET, Primary Care Online Emotion-Regulation Treatment; SSRI, selective serotonin reuptake inhibitor.

^a^ Assessed by the research team using the Mini International Neuropsychiatric Interview for Children and Adolescents, version 6.^15^

^b^ Includes combined, primarily inattentive, and primarily hyperactive-impulsive subtypes.

^c^ Classes based on World Health Organization ATC categories.

### Feasibility and Acceptability Outcomes

The consent rate (the proportion of participants randomized [n = 30] among the number of participants eligible [n = 37]) was 81%. In total, 28 participants (93%) completed at least 1 clinical outcome assessment immediately after treatment. Two participants (7%; 1 in POET [3%]; 1 in supportive treatment [3%]) did not complete either the clinician assessment or the self-report assessment immediately after treatment. Three months after treatment, 26 participants (87%) completed at least 1 clinical outcome assessment. Four participants (13%; 2 in POET [13%]; 2 in supportive treatment [13%]) did not complete either the clinician assessment or the self-report assessment.

Adolescents receiving POET completed a mean (SD) of 4.6 (1.8) of 6 modules, and parents completed a mean (SD) of 5.1 (1.4) modules. Adolescents receiving supportive treatment completed a mean (SD) of 5.7 (0.7) of 6 modules, and parents completed a mean (SD) of 5.9 (0.5). Two of the 30 adolescents (7%; both in POET) did not complete more than 3 modules. All parents completed more than 3 modules.

Adolescents rated treatment credibility and expectancy as satisfactory (mean [SD] CEQ credibility score: POET, 5.79 [1.37]; supportive treatment, 6.33 [1.10]; mean [SD] CEQ expectancy: POET, 49.3% [15.4%]; supportive treatment, 50.0% [16.5%]). Treatment satisfaction was high for both treatments among adolescents (mean [SD] CSQ-8 score: POET, 20.6 [4.93]; supportive treatment, 22.8 [3.12]) and parents (POET, 24.8 [3.56]; supportive treatment, 23.1 [3.76]). Treatment satisfaction did not differ significantly between conditions for adolescents (*P* = .20) or parents (*P* = .25). The mean (SD) therapist time spent delivering POET was 74.7 (57.3) minutes per adolescent and 70.3 (32.1) minutes per parent. The corresponding therapist time for supportive treatment was 64.6 (30.0) minutes per adolescent and 64.1 (29.5) minutes per parent in total. There was no statistically significant difference in therapist time between treatments for adolescents (*P* = .55) or parents (*P* = .59).

Treatment allocation was never revealed to blinded assessors. Blinded assessors’ guesses on treatment allocation were not significantly different from chance either immediately after treatment (67.8% [95% CI, 47.6%-84.1%] correct; *P* = .09) or 3 months after treatment (42.3% [95% CI, 23.4%-63.1%] correct; *P* = .56). Most guesses immediately after treatment (15 of 28 [54%]) were reported to be pure guesses. The rest (13 of 28 [46%]) were based on the participant’s reduction in symptoms or global functioning. The corresponding numbers 3 months after treatment were 24 of 26 (92%) and 2 of 26 (8%), respectively.

Six adverse events were documented, equally distributed between the 2 treatment groups. Events included experiencing increased stress due to the treatment workload and deterioration (eg, sadness and negative thoughts). No serious adverse events were reported.

### Clinical Outcomes

**Figure 2.**
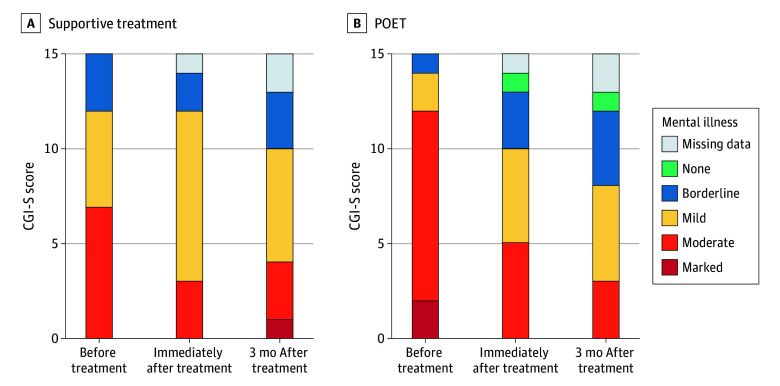
Distribution of Clinical Global Impressions–Severity Scale (CGI-S) Scores at Each Time Point by Treatment Group POET indicates Primary Care Online Emotion-Regulation Treatment.

Results for clinical outcomes are presented in Table 2. The linear quantile mixed-effects model showed a statistically significant reduction in symptom severity for POET (CGI-S: effect size, 1.30; 95% CI, 0.73-1.86) from before treatment to immediately after treatment. This effect was maintained 3 months after treatment (CGI-S: effect size, 1.32; 95% CI, 0.76-1.88). No significant effects were detected for supportive treatment. Figure 2 presents the distribution of CGI-S scores at each time point by treatment group. Immediately after treatment, 7 participants in POET (47%) and 1 participant in supportive treatment (7%) were classified as treatment responders. Three months after treatment, the corresponding numbers for treatment responders were 5 participants in POET (33%) and 3 participants in supportive treatment (20%).

**Table 2.** Results for Clinical Outcomes

Outcome^a^	Score change	Participants, No.^b^	Fixed effects		Effect size (95% CI)^c^
			β (SE)^d^	*P* value	
**Ordinal outcomes**					
Symptom severity, CGI-S score, median (IQR)					
POET					
Before treatment	4.00 (4.00 to 4.00)	15	NA	NA	NA
After treatment	3.00 (2.25 to 4.00)	14	−0.96 (0.21)	<.001	1.30 (0.73 to 1.86)
3 mo After treatment	3.00 (2.00 to 3.00)	13	−0.98 (0.21)	<.001	1.32 (0.76 to 1.88)
Supportive treatment					
Before treatment	3.00 (3.00 to 4.00)	15	NA	NA	NA
After treatment	3.00 (3.00 to 3.00)	14	−0.10 (0.19)	.59	0.13 (0.34 to 0.60)
3 mo After treatment	3.00 (3.00 to 4.00)	13	−0.13 (0.18)	.47	0.16 (0.28 to 0.60)
**Continuous outcomes**					
Symptoms of anxiety and depression, RCADS-47 score, mean (SD)					
POET					
Before treatment	57.53 (12.45)	15	NA	NA	NA
After treatment	42.92 (17.92)	13	−13.32 (4.69)	.009	1.07 (0.37 to 1.84)
3 mo After treatment	41.00 (22.16)	11	−15.98 (4.98)	.003	1.28 (0.51 to 2.08)
Supportive treatment					
Before treatment	54.67 (22.03)	15	NA	NA	NA
After treatment	51.29 (17.84)	14	−3.23 (4.20)	.45	0.15 (−0.24 to 0.53)
3 mo After treatment	44.45 (13.90)	11	−8.14 (4.58)	.09	0.37 (−0.04 to 0.77)
Global functioning, CGAS score, mean (SD)					
POET					
Before treatment	55.20 (5.28)	15	NA	NA	NA
After treatment	61.86 (9.31)	14	6.65 (1.58)	<.001	1.26 (0.66 to 1.85)
3 mo After treatment	63.08 (9.37)	13	8.12 (1.62)	<.001	1.54 (0.95 to 2.14)
Supportive treatment					
Before treatment	57.13 (8.34)	15	NA	NA	NA
After treatment	60.07 (6.16)	14	3.03 (2.79)	.29	0.36 (−0.28 to 1.02)
3 mo After treatment	59.77 (10.69)	13	2.82 (2.85)	.33	0.34 (−0.34 to 1.02)

Abbreviations: CGI-S, Clinical Global Impressions–Severity Scale; CGAS, Children’s Global Assessment Scale; NA, not applicable; POET, Primary Care Online Emotion-Regulation Treatment; RCADS-47, Revised Child Anxiety and Depression Scale.

^a^ Ordinal and continuous outcomes evaluated blinded assessor–rated change from before treatment to immediately after treatment (primary end point) and 3 months after treatment.

^b^ Numbers of participants contributing data.

^c^ For the ordinal measure, effect sizes were estimated by dividing the model estimates of the median effect of time by the SD of the pretreatment outcome. For the continuous measures, Cohen *d* values were calculated by dividing the unstandardized β coefficient for the time variable by the SD of the pretreatment outcome.

^d^ Fixed-effects parameter estimates (β [SE]) represent the effect of time with all randomized individuals (N = 30).

The linear mixed-effects regression analysis showed large significant improvements in symptoms of anxiety and depression for POET (RCADS-47: Cohen *d*, 1.07; 95% CI, 0.37-1.84) from before treatment to immediately after treatment. Results were maintained from before treatment to 3 months after treatment for POET (RCADS-47: Cohen *d*, 1.28; 95% CI, 0.51-2.08). No significant effects were detected for supportive treatment. A large significant improvement was detected in global functioning for POET from before treatment to immediately after treatment (CGAS: Cohen *d*, 1.26; 95% CI, 0.66-1.85) and from before treatment to 3 months after treatment (CGAS: Cohen *d*, 1.54; 95% CI, 0.95-2.14). No improvements were found for supportive treatment.

### Target Mechanism

Results for target mechanisms are presented in Table 3. For POET, large significant improvements were observed for maladaptive cognitive coping from before treatment to immediately after treatment (CERQ-maladaptive: Cohen *d*, 1.10; 95% CI, 0.52-1.70) and from before treatment to 3 months after treatment (CERQ-maladaptive: Cohen *d*, 0.82; 95% CI, 0.22-1.40). No significant effects were detected for supportive treatment. No significant effects were found for adaptive cognitive coping for POET or supportive treatment at either time point. No significant effects were found for self-reported alexithymia for POET or supportive treatment.

**Table 3.** Results for Target Mechanism^a^

Outcome	Score, mean (SD)	Participants, No.^b^	Fixed effects		Cohen *d* (95% CI)^c^
			β (SE)^d^	*P* value	
**Self-reported use of maladaptive cognitive emotion regulation strategies, CERQ-maladaptive**					
POET					
Before treatment	48.40 (10.43)	15	NA	NA	NA
After treatment	36.25 (9.81)	12	−11.48 (3.06)	.001	1.10 (0.52 to 1.70)
3 mo After treatment	39.18 (8.21)	11	−8.52 (3.16)	.01	0.82 (0.22 to 1.40)
Supportive treatment					
Before treatment	45.00 (7.43)	15	NA	NA	NA
After treatment	43.50 (9.28)	14	−1.85 (2.31)	.43	0.25 (−0.35 to 0.85)
3 mo After treatment	41.42 (6.11)	12	−3.50 (2.43)	.16	0.47 (−0.18 to 1.13)
**Self-reported use of adaptive cognitive emotion regulation strategies, CERQ-adaptive**					
POET					
Before treatment	40.87 (8.60)	15	NA	NA	NA
After treatment	42.25 (12.43)	12	0.55 (3.30)	.87	0.06 (−0.72 to 0.84)
3 mo After treatment	42.82 (15.35)	11	2.59 (3.07)	.42	0.21 (−0.29 to 0.71)
Supportive treatment					
Before treatment	44.87 (7.53)	15	NA	NA	NA
After treatment	47.86 (12.78)	14	2.64 (2.41)	.29	0.35 (−0.27 to 0.99)
3 mo After treatment	49.42 (14.22)	12	1.11 (1.36)	.43	0.09 (−0.13 to 0.29)
**Self-reported level of alexithymia, PAQ-S**					
POET					
Before treatment	20.53 (6.13)	15	NA	NA	NA
After treatment	16.25 (7.57)	12	−4.02 (2.18)	.08	0.66 (−0.05 to 1.33)
3 mo After treatment	17.60 (5.74)	10	−2.99 (2.32)	.21	0.49 (−0.25 to 1.22)
Supportive treatment					
Before treatment	20.73 (5.75)	15	NA	NA	NA
After treatment	21.14 (6.51)	14	0.19 (1.60)	.91	−0.03 (−0.57 to 0.51)
3 mo After treatment	21.42 (6.80)	12	−0.03 (1.69)	.99	0.01 (−0.58 to 0.60)

Abbreviations: CERQ, Cognitive Emotion Regulation Questionnaire; NA, not applicable; PAQ-S, Perth Alexithymia Questionnaire–Short Form; POET, Primary Care Online Emotion-Regulation Treatment.

^a^ Ordinal and continuous outcomes evaluating change from before treatment to immediately after treatment (primary end point) and 3 months after treatment.

^b^ Numbers of participants contributing data.

^c^ Estimated mean change from before treatment relative to the SD before treatment.

^d^ Fixed-effects parameter estimates (β [SE]) represent the effect of time with all randomized individuals (N = 30).

## Discussion

This single-blind randomized clinical trial found that a brief online emotion regulation treatment and the active control treatment were acceptable and feasible for adolescents with mental health problems. The high consent and assessment completion rates support the feasibility of the study. Adherence to treatments and ratings of treatment credibility, expectancy, and satisfaction with treatment support acceptability of both treatments. These findings, combined with the efficient use of therapist time, feasibility of the blinding procedure, and limited adverse events, are comparable to results from previous studies on online emotion regulation treatments.^25,26,35^ Adolescents receiving POET, but not supportive treatment, showed decreases in symptom severity and symptoms of depression and anxiety and improvement in global functioning immediately after treatment and 3 months after treatment. A decrease in maladaptive cognitive coping was observed in POET but not in supportive treatment. For other target mechanism measures, no effect was found at any time point. These results provide initial evidence supporting the feasibility, acceptability, and potential efficacy of POET as a brief online treatment for adolescents with mental health problems.

The finding that a brief intervention targeting emotion regulation improved clinical outcomes and emotion regulation is consistent with research on youth emotion regulation interventions, both in person^11,13^ and online.^25,26,36^ Unlike previous online interventions,^25^ POET comprised 6 modules with limited weekly therapist involvement. These findings are encouraging, indicating that a brief, scalable intervention can influence important outcomes in primary care.

### Strengths and Limitations

This study has several strengths. These include the randomized design, the use of an active control treatment, and the integrity of the blinded assessments.

The study also has limitations. Most of the participants were self-referred, which introduced a potential selection bias, as participants in this study may have been more motivated to engage in treatment than the typical adolescent patient. However, 80% of the participants had previous contact with health care for mental health problems, indicating that the sample was clinically representative. Even though the a priori feasibility criteria for completion of assessments were fulfilled in this trial, future studies of POET should make further efforts to boost the completion rate of adolescent assessments. The goal at this early stage of treatment development was to compare feasibility with a credible active control matched in length, structure, clinician attention, monitoring, and symptom reporting to maximize internal validity. The active control condition allows for a more precise evaluation of the intervention while avoiding the variability inherent in routine care. However, this design limits the clinical interpretation compared with using a control group receiving treatment as usual. Additionally, POET was not compared with a gold standard treatment, which further restricts conclusions about its relative clinical effectiveness. The small sample size and the lack of stratification in the randomization procedure are shortcomings of the study that prevented between-group analysis of effectiveness. Future studies should examine the effectiveness of POET with a larger, more gender-diverse sample. Furthermore, if long-term effectiveness is confirmed in a definitive randomized clinical trial, an implementation study would be appropriate to assess whether POET is noninferior to the gold standard treatment while incorporating a health economic evaluation.

## Conclusions

The results of this single-blind randomized clinical trial demonstrated that a brief online emotion regulation treatment requiring limited therapist time per patient was associated with reductions in clinical mental health symptoms and maladaptive cognitive coping strategies. Given that adolescents represent a large patient group with limited access to psychological treatment, these findings suggest that POET is a promising treatment in primary care, with the potential for broad outreach and improved accessibility for adolescents with mental health problems.

## Notes

Supplement 1.
Trial Protocol

Supplement 2. **eAppendix.** List of Measures **eTable 1.** Content of the Adolescent Program in POET Treatment **eTable 2.** Content of the Parent Course in POET Treatment **eTable 3.** Content of the Adolescent Program in Supportive Treatment **eTable 4.** Content of the Parent Course in Supportive Treatment **eFigure 1.** Screenshot of a Worksheet From Primary Care Online Emotion-Regulation Treatment (POET) **eFigure 2.** Screenshot of the Message Function of Both Treatments **eFigure 3.** Screenshot of a Worksheet From the Active Control Treatment
eReferences

Supplement 3.
Data Sharing Statement
