# Supplementary material for: Online Transdiagnostic Emotion Regulation Treatment for Adolescents With Mental Health Problems: A Randomized Clinical Trial
Source: JAMA Netw Open. 2025 Jun 11;8(6):e2514871. doi: 10.1001/jamanetworkopen.2025.14871 (PMC12159777; doi:10.1001/jamanetworkopen.2025.14871)
Supplement: Supplement 1. — Trial Protocol [file jamanetwopen-e2514871-s001.pdf]

Study Trial Protocol

Online Transdiagnostic Emotion Regulation Treatment for Adolescents with Mental Health Problems: A Randomized Feasibility Trial

Trial ID:	NCT05032547 (ClinicalTrials.gov ID)<br><br>2022-01053-01(approval no. Swedish Ethical Review Authority)
Version number:	3
Date:	2024-12-18
Sponsor:	Region Stockholm
Sponsor representative:	Kersti Ejeby
Co-sponsor:	Karolinska institutet
Co-sponsor representative:	Johan Bjureberg

Table of content

REVISION HISTORY **3**
CONTACT INFORMATION **3**
BACKGROUND **5**
AIM AND OBJECTIVES **5**
Aim 5
Primary objective 5
Secondary objective 6
Research questions 6
Time points 6
TRIAL DESIGN AND PROCEDURES **6**
Overall trial design 6
Flow chart 7
Start and end of the clinical trial 7
Subject selection 7
Inclusion criteria 7
Exclusion criteria 7
Screening and inclusion 8
Withdrawal criteria 8
TREATMENTS **8**
The active treatment 8
The active control treatment 9
Therapist support 9
Randomization 9
Blinding 9
MEASURES **10**
Safety procedures 12
Handling of Adverse Events 12
ETHICS **13**
STATISTICAL ANALYSES **13**
Power calculations 13
COLLECTION, HANDLING, AND ARCHIVING OF DATA **13**
REFERENCES **15**

Revision history

Protocol version	Date of Issue	Summary of changes
1	2022-01-10	Included the questionnaire Perth Alexithymia Questionnaire (PAQ) to measure Alexithymia.<br>Included assessments three-month post-treatment.<br>Expanded the numbers of exclusion criteria before study commenced.
2	2023-08-30	Decided not to analyze and report for main outcome paper:<br>- Weekly measures of Revised Child Anxiety and Depression Scale and the Cognitive Emotion Regulation Questionnaire.<br>- Strengths and Difficulties Questionnaire.<br>- Brief 16-item Version of the Difficulties in Emotion Regulation.<br>- Emotion Regulation Questionnaire Child/Adolescents Version.<br>- Beliefs About Emotions Questionnaire<br>- Emotion Regulation Diversity Index.
3	2025-03-2	Statistical analysis was updated in response to reviewers' comments and the decision to remove all between group analysis and only present within group analysis separately for the two intervention groups.

Contact information

Responsibility in the clinical trial	
Sponsor representative	Kersti Ejeby, Licensed medical doctor<br>Gustavsbergs vårdcentral<br>Odelbergs väg 19D, 134 40 Gustavsberg, Sweden
Principal Investigators	Johan Bjureberg, Associate professor<br>Karolinska institutet<br>Norra stationsgatan 69, plan 7<br>113 64 Stockholm<br>Johan.bjureberg@ki.se

95

Background

Mental health problems in youth are a global problem, causing incalculable suffering in youth and families, harming long-term prospects of youths, and creating substantial economic costs to society.[1] Although treatments exist, these treatments are not fully addressing these problems for several reasons. First, although many youth in need of treatment for mental health problems do not meet criteria for any mental health disorder[2,3] or they meet criteria for several disorders[4], most treatments focus on a subset of mental disorders and do not address subthreshold or multi-disorder mental health problems. Second, available treatments do not typically target transdiagnostic disease mechanisms. Third, the available treatments are limited in efficacy.[5] Fourth, most youth in need do not receive treatment due to social stigma and structural barriers such as geographical distance to treatment providers.[6] Of particular concern is the lack of attention to subthreshold or multi-disorder mental health problems.[3,4] There is a lack of evidence-based treatment protocols for patients without a formal disorder or fulfilling criteria for several disorders – a problem that has recently been identified within Swedish primary mental health care.[7] Many leading researchers and institutions have suggested that progress within mental health research and treatment requires a shift away from a focus on specific mental disorders to a more transdiagnostic perspective, addressing underlying processes.[8] One promising observation in this direction is the robust finding that the frequency of usage of adaptive and maladaptive emotion regulation strategies is related to a wide range of mental health problems, suggesting that emotion regulation may be an important treatment target.[9] Indeed, a meta-analysis indicates that interventions targeting emotion regulation in youth can improve mental health problems.[10] However, the authors highlight that there is limited evidence with respect to interventions targeting emotion regulation in different age groups and psychopathologies, as well as a lack of longitudinal studies investigating the temporal relationship between emotion regulation and psychopathology. The authors conclude that appropriately sized randomized controlled trials (RCT) evaluating scalable transdiagnostic treatments targeting emotion regulation are urgently needed to assess both how well they work in youth (i.e., improve mental health) and the mechanisms by which they work (e.g., through improved emotion regulation).

Aim and objectives

Aim

The overall aim of the project is to study feasibility, acceptability and preliminary effects on clinical outcome and target mechanism of an online transdiagnostic emotion regulation treatment called Primary care Online Emotion-regulation treatment (POET).

Primary objective

Primary objective of this trial is to investigate the feasibility and acceptability of POET relative to an active control treatment.

Secondary objective

Secondary objectives are to investigate the preliminary effectiveness on clinical outcomes and target mechanism of POET relative to an active control treatment.

Research questions

1. Are study procedure and design feasible, operationalized as (1) consent rate, defined as the proportion of eligible participants who consented to inclusion and randomization; and (2) completion of assessments, defined as completing at least one clinical outcome immediately post-treatment?
2. Are POET treatment and the active control treatment acceptable, operationalized as (1) compliance with the treatments, defined as completing at least three of six online modules; (2) youth and parent ratings of credibility and expectancy of treatment after completing the first module; and (3) youth and parent satisfaction with treatment/course?
3. Is participation in POET, relative to participation in the active control treatment, associated with reduction in clinical outcomes, operationalized as (1) symptoms of anxiety and depression, (2) global functioning, and (3) symptom severity and improvement, and how large is the effect?
4. Is participation in POET, relative to participation in the active control treatment, associated with a reduction in maladaptive emotion regulation strategies and alexithymia and an increase in adaptive emotion regulation strategies, and how large is the effect?

Time points

The primary endpoint of this trial is immediately post-treatment. The secondary endpoint of this trial is three-month post-treatment.

Trial design and procedures

Overall trial design

This single-blinded, randomized feasibility trial, targeting adolescents with mental health problems compares POET, an online transdiagnostic emotion regulation treatment, to an active control treatment. Participants are followed up immediately post-treatment and three-month post-treatment.

Flow chart

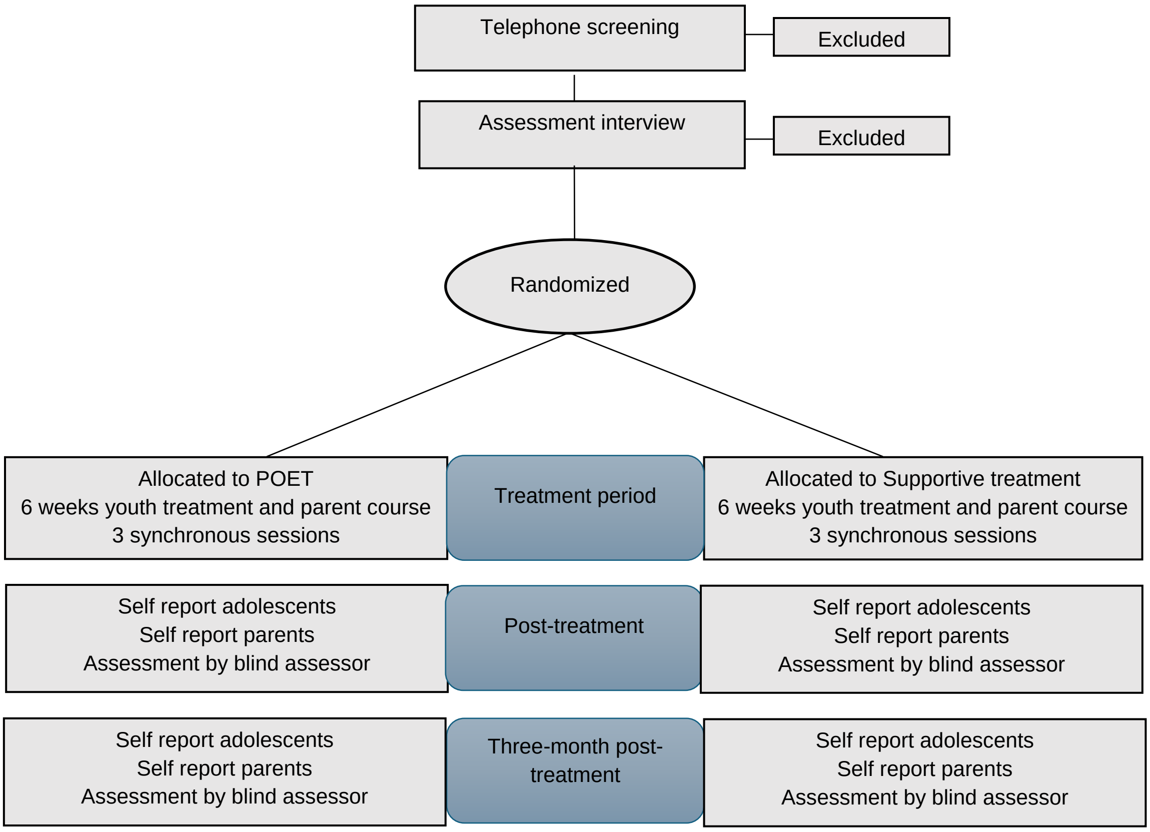

Start and end of the clinical trial

The start of the trial is defined as when the first participant is randomized. The trial ends when the last subject has completed the last three-month post-treatment

Subject selection

Participants (adolescents and one parent) are recruited through self-referrals and referrals from healthcare professionals. The trial is advertised within Primary care services and on social media.

Inclusion criteria

- Adolescent between the age of 12 to 17 years
- Presenting with mental health problems
- Having at least one parent willing to participate in the parent course.

Exclusion criteria

- Severe mental illness that required specialized care or low global functioning corresponding to a Children’s Global Assessment Scale (CGAS;)15 score < 41
- Acute suicidality
- Ongoing psychological treatment
- Changes in psychopharmacological medication during the past 2 months
- Insufficient skills in speaking and understanding Swedish
- Life circumstances that could prevent treatment participation (e.g., domestic violence).

Screening and inclusion

Individuals can access information about the study via a study website or via Primary care services in Stockholm, Sweden. People interested in participating in the study can make a self-referral via the self-assessment tool and website. When potential participants have been referred to the study or have made a self-referral, members of the research project group will contact the parents and give information about the study. If parents and adolescents are interested in participating, they are invited to a digital clinical assessment. Parents and adolescents answer self-assessment measures and sign informed consent before the clinical assessment. Participants between 12 to 14 years old provide verbal consent, while both caregivers, if applicable, provide written consent. Participants between 15 to 17 years old provide written consent themselves. During the assessment interview, the inclusion and exclusion criteria are assessed through clinician-administered interviews and assessments conducted by a licensed psychologist. Hence, eligibility is established before randomization. Individuals who meet the inclusion criteria and no exclusion criteria are offered participation in the study. Excluded participants receive treatment recommendations if needed.

Withdrawal criteria

Subjects can discontinue their participation in the trial at any time.

Treatments

Treatments in both conditions will be delivered in a blended treatment format combining asynchronous therapist-guided online modules (text/videos/audio/messaging function) with synchronous sessions delivered over video-link. Both treatments will include six treatment modules for the youth and parents respectively, administered over six weeks. Both treatments will be delivered on a secure online platform. The family participates in a synchronous session before starting the treatment and immediately after completing the final module and the last module. These sessions are designed to provide practical information about the treatment platform, motivate participants, define treatment goals (only in the POET treatment), and assess if further care is required after the treatment.

The active treatment

POET will be adapted from online Emotion Regulation Individual Therapy for Adolescents developed by the research group[11–14]. The main changes will include (1) revisions to make psychoeducation and examples appropriate for youth with mild to moderate mental health problems and less focus on self-harm; (2) reduced treatment length from 12 to 6 weeks; and (3) the inclusion of synchronous video-link meeting. The adaptations are based on prior experience of the target population and qualitative interviews with prior study participants.[15] The theoretical foundation of the POET treatment draws from the Extended Process Model of Emotion Regulation.[16] The youth component of POET will include psychoeducation about emotions and skill training, focusing on five emotion regulation strategies aligned with the emotion-

generative sequence of situation, attention, appraisal, and response. The parent course focuses on how parents can support the adolescent in using more adaptive and fewer maladaptive emotion regulation strategies and skill training in effectively responding to their children's and their own emotions.

The active control treatment

The active control will be supportive treatment. This treatment is designed to control for effects of non-specific treatment components that are common to most psychosocial interventions and that are known to be associated with reduction of symptoms, such as receiving general counseling, attention from a therapist, and monitoring of one's emotions and behaviors. The active control treatment is an online Supportive treatment based on an online supportive treatment previously evaluated for social anxiety disorder. [17] It includes psychoeducation on mental health problems and information on five themes related to adolescent well-being (school, family, friends, self-esteem, and social support) Participants are instructed to reflect upon each theme throughout the treatment. None of the active elements of the experimental treatment were included in the active control treatment. The parental course includes weekly reflections on how to support their adolescents' well-being.

Therapist support

Throughout treatment, adolescents and parents have regular contact with an assigned therapist through a secure online platform, where the treatment content is also presented. The therapist provides feedback, assists in planning homework assignments, and is available for questions at any time during treatment. The therapists are licensed psychologists with extensive experience in treating adolescents with mental health problems. Therapists will provide both the POET treatment and the active control treatment after careful training in both methods. Therapists will receive weekly supervision.

Randomization

Eligible participants will be randomly allocated (1:1) to POET treatment or Supportive treatment. Randomization will be conducted by an independent researcher using a true random number service. The random allocation sequence will be placed in opaque sealed envelopes.

Blinding

Assessors are blinded to treatment allocation, and all participants received explicit instructions not to reveal their treatment condition to the blinded assessor. The integrity of the blinding is assessed by having blinded assessors guess the group allocation following each assessment and specify the reason for their guess, choosing between 1) the family accidentally revealed the condition, 2) symptom reduction, 3) global functioning, or 4) a pure guess. Blind assessors are also asked to report whether the group allocation was accidentally disclosed.

Measures

Outcome measures include both blinded assessor-rated telephone interviews and online self-reported assessments immediately after post-treatment and three-month post-treatment. Data, in the form of clinician- and self-rated assessments, will be collected for all participants pre-treatment, immediately post-treatment, and at three-month post-treatment. An independent rater blind to treatment condition will conduct the immediate post-treatment assessment and the three-month post-treatment assessments.

Screening measures

-Psychiatric comorbidity: This will be clinically assessed with Mini-Kid International Neuropsychiatric Interview, version 6. (MINI KID, [18]).

-Suicidality: One question that aims to screen for and identify suicidality will be administered weekly to all adolescents.

Outcomes for research question 1: (feasibility measures):

The proportion of eligible participants who consent to inclusion and randomization will be calculated.

The proportion of completed assessments (at least one clinical outcome) immediately post-will be calculated.

Outcomes for research question 2: (acceptability measures)

The proportion of participants who complete at least three of six online modules will be calculated.

The Credibility/Expectancy Questionnaire (CEQ, [19]) will be administered to adolescents and parents after module 1 when they have been introduced to the treatment rationale.

Client Satisfaction Questionnaire (CSQ,[20]). This questionnaire will be administered to all participants immediately post-treatment.

Outcomes for research question 3: (clinical outcomes measures)

The Revised Child Anxiety and Depression Scale (RCADS, [21]) will be used to measure symptoms of anxiety and depression. This questionnaire will be administered by a clinician at pre-treatment and by a clinician blinded to treatment allocation immediately post-treatment and at three-month post-treatment.

Childrens´ Global Assessment Scale (C-GAS,[14]) will be used to measure global functioning. A clinician will rate global functioning at pre-treatment and by a clinician blinded to treatment allocation immediately post-treatment and at three-month post-treatment.

The Clinical Global Impressions -Severity and Improvement Scale (CGI-S/I,[22]) will be used to measure symptom severity and improvement. A clinician will rate symptom

severity at pre-treatment and a clinician blinded to treatment allocation will rate symptom severity and improvement immediately post-treatment and at three-month post-treatment. Symptom severity and improvement are rated on a single item ranging from 1-7, with higher scores indicating more severe symptoms and lower scores indicating more improvement.

Outcomes for research question 4 (target mechanism measures)

The Cognitive Emotion Regulation Questionnaire (CERQ,[23]) will be used to measure emotion regulation strategies. This questionnaire will be administered to adolescents pre-treatment, immediately post-treatment, and at three-month post-treatment.

Perth Alexithymia Questionnaire (PAQ,[24]) will be used to measure alexithymia. This questionnaire will be administered to adolescents pre-treatment, immediately post-treatment, and at three-month post-treatment.

Other measures:

Demographic background data: Parents will answer questions about the parental educational level, occupational status, prior treatment history, and information on how the participants got in contact with the study will be registered (e.g., via self-referral or referral from health care).

Adverse events: Participants are asked to report and rate the discomfort of the eventual adverse events caused by their participation in the treatment. These questions will be administered to all participants at post-treatment. Clinicians will also report eventual adverse events during the treatment period.

Therapist time: The total therapist time spent on both reading what adolescents and parents have written in the modules and providing feedback and messaging participants will be calculated.

	Pre assessment	Post module 1	Post-treatment	3-months post-treatment
**Clinician-rated (blinded)**	RCADS<br>C-GAS<br>CGI-S/I		RCADS<br>C-GAS<br>CGI-S/I	RCADS<br>C-GAS<br>CGI-S/I
**Adolescent- rated**	CERQ<br>PAQ	CEQ	CERQ<br>PAQ<br>CSQ<br>ADVERSE EVENTS	CERQ<br>PAQ
**Parent-rated**	Questions about demographic background	CEQ	CSQ	

Table 1: List of measures and time-points

Safety procedures

During the study period, the adolescents will be under continuous monitoring by the research group. At inclusion, an experienced clinician will assess suicide risk. Patients with acute suicidality will be excluded from the study and receive treatment recommendation and referral if needed. Suicidal ideation will be continuously monitored (weekly during treatment and at post-treatment and three-month post-treatment), ensuring that sudden deterioration in mental health is detected and additional care is provided.

Handling of Adverse Events

Adverse Event (AE) is defined as: Any untoward medical occurrence in a subject to whom a medicinal product is administered and which does not necessarily have a causal relationship with this treatment.

Serious Adverse Event (SAE) is defined as: Any untoward medical occurrence that at any dose requires inpatient hospitalization or prolongation of existing hospitalization, results in persistent or significant disability or incapacity, is life-threatening, or results in death.

During treatment, AE and SAR are registered by a clinician. All AE that occurs during the trial and which are observed by a clinician or reported by the subject will be registered regardless of whether they are assessed as related to the treatment of not. Assessment of causal relationship, severity, and whether the AE is considered to be an SAE will be made by the project investigator. For each AE/SAE, a

description of the event is recorded, start and stop dates, causal relationship, severity, and whether it is an AE or SAE. Serious Adverse Events (SAE) are reported to the sponsor on a special SAE form within 24 hours of the investigator being informed of the SAE.

Ethics

The trial will be performed in compliance with this protocol, the Declaration of Helsinki, ICH-GCP (Good Clinical Practice), and current Swedish regulations governing this clinical trial. This study has been approved by the Swedish Ethical Review Authority.

Statistical analyses

The data will be visually inspected using histograms to check for distribution assumptions and outliers. The analyses will apply intention-to-treat (ITT) method.

Descriptive statistics, independent sample t-test, and binomial test will be used to analyze primary objective and research questions 1-3. Differences between treatments in satisfaction and therapist time will be analyzed using independent sample t-tests. To test whether blind assessors’ guesses on treatment allocation differed from chance, a binomial test will be used.

Within group effects will be evaluated in an exploratory manner according to the intention-to-treat principle. Linear-mixed effects regression for continuous outcomes and linear quantile mixed models for ordinal outcomes were fitted for the POET and the Supportive groups separately. These include a random intercept for each participant, and the dummy coded time variable (pre-treatment, post-treatment, and three-month post-treatment, with the pre-treatment time point as reference category. Median effects were estimated, using a linear quantile mixed model using the lqmm[31] package in R. P-values and CI were calculated using the bootstrap procedure implemented in the lqmm package, with 1000 replications. For CGI-I number of participants classified as responders are presented. Treatment response was defined as a CGI-I rating of 1 (very much improved) or 2 (much improved)

Power calculations

30 participants will be included in this study, a sufficient number of participants to answer the research questions about feasibility and acceptability. The sample size (N = 30), given an estimate of 20% dropout, gives a power of 80% to detect a meaningful standardized average difference between pre- and post-measurement in each condition of d = 0.6 given alpha level .05.

Collection, handling, and archiving of data

All individuals handling personal data are bound by confidentiality agreements and/or professional secrecy obligations through their employment terms or confidentiality

agreements in accordance with the sponsor. Each study participant's personal data will be protected by confidentiality, and participants will be given a number as their identifier. All correspondence between participants and therapists will take place on platforms which require two-factor authentication. The code key is stored in a security-classified database for sensitive personal data within the organization of the sponsor. Data is stored in accordance with the sponsors archival rules for research documents.

Assessments are administered via the internet and all answers, from participants and clinicians, are securely stored on a database developed for this purpose and have been used in several completed and ongoing studies. All data traffic is encrypted, ensuring a high security level.

References

1. Patton GC, Sawyer SM, Santelli JS, Ross DA, Afifi R, Allen NB, et al. Our future: a Lancet commission on adolescent health and wellbeing. The Lancet. 2016 Jun;387(10036):2423–78.

2. Roberts RE, Fisher PW, Blake Turner J, Tang M. Estimating the burden of psychiatric disorders in adolescence: the impact of subthreshold disorders. Soc Psychiatry Psychiatr Epidemiol. 2015 Mar 1;50(3):397–406.

3. Lewinsohn PM, Shankman SA, Gau JM, Klein DN. The prevalence and co-morbidity of subthreshold psychiatric conditions. Psychol Med. 2004 May;34(4):613–22.

4. Merikangas KR, He J ping, Burstein M, Swanson SA, Avenevoli S, Cui L, et al. Lifetime Prevalence of Mental Disorders in US Adolescents: Results from the National Comorbidity Study-Adolescent Supplement (NCS-A). J Am Acad Child Adolesc Psychiatry. 2010 Oct;49(10):980–9.

5. Marchette LK, Weisz JR. Practitioner Review: Empirical evolution of youth psychotherapy toward transdiagnostic approaches. J Child Psychol Psychiatry. 2017;58(9):970–84.

6. Radez J, Waite P, Chorpita B, Creswell C, Orchard F, Percy R, et al. Using the 11-item Version of the RCADS to Identify Anxiety and Depressive Disorders in Adolescents. Res Child Adolesc Psychopathol. 2021;49(9):1241–57.

7. Första linjen för barns och ungas psykiska hälsa 21/22 [Internet]. Stockholm: Swedish Association of Local Authorities and Regions; 2022 [cited 2024 Sep 4]. Available from: https://www.uppdragpsykiskhalsa.se/wp-content/uploads/2022/06/La%CC%88gesrapport-Fo%CC%88rsta-linjen-2022_final_.pdf

8. Insel T, Cuthbert B, Garvey M, Heinssen R, Pine DS, Quinn K, et al. Research Domain Criteria (RDoC): Toward a New Classification Framework for Research on Mental Disorders. Am J Psychiatry. 2010 Jul;167(7):748–51.

9. Sheppes G, Suri G, Gross JJ. Emotion Regulation and Psychopathology. Annu Rev Clin Psychol. 2015;11(1):379–405.

10. Moltrecht B, Deighton J, Patalay P, Edbrooke-Childs J. Effectiveness of current psychological interventions to improve emotion regulation in youth: a meta-analysis. Eur Child Adolesc Psychiatry. 2021 Jun;30(6):829–48.

11. Bjureberg J, Sahlin H, Hellner C, Hedman-Lagerlöf E, Gratz KL, Bjärehed J, et al. Emotion regulation individual therapy for adolescents with nonsuicidal self-injury disorder: a feasibility study. BMC Psychiatry. 2017 Dec 28;17(1):411.

12. Bjureberg J, Sahlin H, Hedman-Lagerlöf E, Gratz KL, Tull MT, Jokinen J, et al.

Extending research on Emotion Regulation Individual Therapy for Adolescents (ERITA) with nonsuicidal self-injury disorder: open pilot trial and mediation analysis of a novel online version. BMC Psychiatry. 2018 Oct 11;18(1):326.

13. Bjureberg J, Ojala O, Hesser H, Häbel H, Sahlin H, Gratz KL, et al. Effect of Internet-Delivered Emotion Regulation Individual Therapy for Adolescents With Nonsuicidal Self-Injury Disorder: A Randomized Clinical Trial. JAMA Netw Open. 2023 Jul 13;6(7):e2322069.

14. Shaffer D, Gould MS, Brasic J, Ambrosini P, Fisher P, Bird H, et al. A Children's Global Assessment Scale (CGAS). Arch Gen Psychiatry. 1983 Nov 1;40(11):1228–31.

15. Simonsson O, Engberg H, Bjureberg J, Ljótsson B, Stensils J, Sahlin H, et al. Experiences of an Online Treatment for Adolescents With Nonsuicidal Self-injury and Their Caregivers: Qualitative Study. JMIR Form Res. 2021 Jul 23;5(7):e17910.

16. Gross JJ. Emotion Regulation: Current Status and Future Prospects. Psychol Inq. 2015 Jan 2;26(1):1–26.

17. Nordh M, Wahlund T, Jolstedt M, Sahlin H, Bjureberg J, Ahlen J, et al. Therapist-Guided Internet-Delivered Cognitive Behavioral Therapy vs Internet-Delivered Supportive Therapy for Children and Adolescents With Social Anxiety Disorder: A Randomized Clinical Trial. JAMA Psychiatry. 2021 Jul 1;78(7):705–13.

18. Sheehan DV, Sheehan KH, Shytle RD, Janavs J, Bannon Y, Rogers JE, et al. Reliability and Validity of the Mini International Neuropsychiatric Interview for Children and Adolescents (MINI-KID). J Clin Psychiatry. 2010 Mar 15;71(03):313–26.

19. Devilly GJ, Borkovec TD. Psychometric properties of the credibility/expectancy questionnaire. J Behav Ther Exp Psychiatry. 2000 Jun 1;31(2):73–86.

20. Larsen DL, Attkisson CC, Hargreaves WA, Nguyen TD. Assessment of client/patient satisfaction: Development of a general scale. Eval Program Plann. 1979 Jan 1;2(3):197–207.

21. Chorpita BF, Yim L, Moffitt C, Umemoto LA, Francis SE. Assessment of symptoms of DSM-IV anxiety and depression in children: a revised child anxiety and depression scale. Behav Res Ther. 2000 Aug 1;38(8):835–55.

22. Busner J, Targum SD. The clinical global impressions scale: applying a research tool in clinical practice. Psychiatry Edgmont Pa Townsh. 2007 Jul;4(7):28–37.

23. Garnefski N, Kraaij V. Cognitive emotion regulation questionnaire – development of a short 18-item version (CERQ-short). Personal Individ Differ. 2006 Oct 1;41(6):1045–53.

24. Preece DA, Mehta A, Petrova K, Sikka P, Bjureberg J, Chen W, et al. The Perth

Alexithymia Questionnaire-Short Form (PAQ-S): A 6-item measure of alexithymia. J Affect Disord. 2023 Mar 15;325:493–501.

25. Rubin DB. Inference and Missing Data. ETS Res Bull Ser. 1975;1975(1):i–19.

26. Geraci M. Linear Quantile Mixed Models: The lqmm Package for Laplace Quantile Regression. J Stat Softw. 2014 May 6;57:1–29.
