# Supplementary material for: Online Transdiagnostic Emotion Regulation Treatment for Adolescents With Mental Health Problems: A Randomized Clinical Trial
Source: JAMA Netw Open. 2025 Jun 11;8(6):e2514871. doi: 10.1001/jamanetworkopen.2025.14871 (PMC12159777; doi:10.1001/jamanetworkopen.2025.14871)
Supplement: Supplement 2. — eAppendix. List of Measures eTable 1. Content of the Adolescent Program in POET Treatment eTable 2. Content of the Parent Course in POET Treatment eTable 3. Content of the Adolescent Program in Supportive Treatment eTable 4. Content of the Parent Course in Supportive Treatment eFigure 1. Screenshot of a Worksheet From Primary Care Online Emotion-Regulation Treatment (POET) eFigure 2. Screenshot of the Message Function of Both Treatments eFigure 3. Screenshot of a Worksheet From the Active Control Treatment eReferences [file jamanetwopen-e2514871-s002.pdf]

Supplementary Online Content

Sjöblom K, Frankenstein K, Klintwall L, et al. Online transdiagnostic emotion regulation treatment for adolescents with mental health problems: a randomized clinical trial. *JAMA Netw Open*. 2025;8(6):e2514871. doi:10.1001/jamanetworkopen.2025.14871

eAppendix. List of Measures
eTable 1. Content of the Adolescent Program in POET Treatment
eTable 2. Content of the Parent Course in POET Treatment
eTable 3. Content of the Adolescent Program in Supportive Treatment
eTable 4. Content of the Parent Course in Supportive Treatment
eFigure 1. Screenshot of a Worksheet From Primary Care Online Emotion-Regulation Treatment (POET)
eFigure 2. Screenshot of the Message Function of Both Treatments
eFigure 3. Screenshot of a Worksheet From the Active Control Treatment
eReferences

This supplementary material has been provided by the authors to give readers additional information about their work.

© 2025 Sjöblom K et al. *JAMA Network Open*.

eAppendix. List of measures

Diagnostic Assessments
A full diagnostic interview was conducted by a licensed psychologist using the MINI-KID International Neuropsychiatric Interview, version 6. [1]

Clinical outcomes

Symptoms of depression and anxiety were measured using The Revised Child Anxiety and Depression Scale (RCADS).[2] This scale has 47 items, total score ranging from 0 to 141, with higher scores indicating more severe symptoms. This scale was clinician-administered before randomization and administered by a blinded assessor at immediately post-, and three-month post-treatment. At the end of this measure, adolescents also answered a question regarding suicidal ideation during the past week, where participants could choose any of the following answers: "*I am not thinking about killing myself*", "*I am thinking about killing myself, but would never do it*", and "*I want to kill myself*".

Symptom severity was measured using The Clinical Global Impressions - Severity scale (CGI-S).[3] Symptom severity is rated on a single item ranging from 1-7, with higher scores indicating more severe symptoms. This scale was clinician-administered before treatment and administered by a blinded assessor at post-, and three-months post-treatment.

Symptom improvement was measured using The Clinical Global Impressions - Improvement scales (CGI-I).[3] Symptom improvement is rated on a single item ranging from 1-7, with lower scores indicating more improvement. This scale was administered by a blinded assessor at immediately post-, and three-months post-treatment.

Global impairment was measured using the Children's Global Assessment Scale (CGAS).[4] Scores on this measure range from 1-100, with higher scores indicating better global functioning. This scale was clinician-administered before treatment and administered by a blinded assessor at immediately post-, and three-month post-treatment.

Cognitive emotion regulation strategies were measured using The Cognitive Emotion Regulation Questionnaire (CERQ-short).[5] This scale consists of nine subscales that can be divided into maladaptive and adaptive cognitive strategies.[6] This scale has 18 items, total subscale score ranges from 4-20. The higher the subscale score, the more a specific cognitive strategy is used. This measure was self-rated by adolescents at pre-treatment, immediately post-, and three-month post-treatment.

Alexithymia was measured using The Perth Alexithymia Questionnaire-Short Form (PAQ-S).[7] This scale has 6 items, the total score ranges from 6 to 42, with higher scores indicating higher levels of alexithymia. This measure was self-rated by adolescents at pre-treatment, immediately post-, and three-month post-treatment

Treatment credibility and expectancy were measured with The Credibility/Expectancy Questionnaire (CEQ).[8] Following previous research [9], an overall credibility rating was calculated by calculating the average of the first three items of the CEQ (range 3-27). Expectancy was measured by item four "*By the end of the therapy period, how much improvement in your anxiety do you think will occur?*" (range 0-100 in 10-point increments). Higher scores indicate greater credibility and expectancy. This measure was self-rated by adolescents before module two.

Satisfaction with treatment was measured using the Client Satisfaction Questionnaire (CSQ).[10] This scale has 8 items ranging from 8-32 points with higher scores indicating greater treatment satisfaction. This measure was rated by adolescents and parents immediately post-treatment.

At immediately post-treatment, adolescents were asked to report and describe any potential adverse events that occurred during the treatment period. They were asked to

© 2025 Sjöblom K et al. *JAMA Network Open*.

rate the discomfort of eventual events, both at the time of occurrence and currently, on a scale from 0 (did not affect me at all) to 3 (affected me very negatively).

© 2025 Sjöblom K et al. *JAMA Network Open*.

eTable 1. Content of the adolescent program in POET Treatment

**Module**	**Name and theme of module**	**Content**
Module 1	Understanding how emotions work	• Psychoeducation about emotions, functionality of emotions, and emotion regulation and their relationship to mental health problems.<br>• Introduction to fictive example characters.<br>• Introduction to the Process model of emotion regulation.<br>• **Homework**: Register emotions, label them as helpful/unhelpful, and identify vulnerability/resilience factors.
Video-link session:	Follow-up session	• Goal formulation (specific, measurable, assignable, realistic, and time-related goals).<br>• Address any motivational problems.<br>Addressing adolescent´s vulnerability/resilience factors.
Module 2	Feeling better by changing or modifying the situation	• Repetition module 1 and review homework.<br>• Addressing unhelpful avoidance.<br>• Information on how to stay in or seek out situations that are avoided and how to seek out positive situations.<br>• **Homework**: Reduce vulnerability factor, stay in situations that are avoided or seek out positive situation.
Module 3	Feeling better by shifting your focus	• Repetition module 2 and review homework.<br>• Psychoeducation about the components of emotions and addressing emotional awareness.<br>• Psychoeducation on shifting one's focus through distraction or by identifying one´s emotions.<br>**Homework:** Practicing distraction and identification of emotions.
Module 4	Feeling better by letting go of or changing your thoughts	• Repetition module 3 and review homework.<br>• Psychoeducation about thoughts, addressing common negative beliefs about emotions and emotion regulation.<br>• Psychoeducation about mindful observations, acceptance of thoughts, and helpful appraisal styles.<br>• **Homework:** Practicing mindful observation, acceptance of thoughts, and helpful appraisal styles.
Module 5	Feeling better by changing your behaviors	• Repetition module 4 and review homework.<br>• Psychoeducation about behaviors and impulses, addressing impulsivity.<br>• Engage in alternative behaviors in situations when one typically acts impulsively.<br>**Homework:** Engage in alternative behaviors instead of engaging in impulsivity.
Module 6	Summary	• Repetition module 4 and review homework.<br>• Summary and maintenance plan.

© 2025 Sjöblom K et al. *JAMA Network Open*.

eTable 2. Content of the parent course in POET Treatment

Module	Name and theme of module	Content
Module 1	Emotions and emotion regulation	• Introduction to emotion regulation and the role of parents during the treatment period.<br>• Introduction to fictive parent characters.<br>• **Homework**: Supporting the adolescent handling vulnerability/resilience factors.
Video-link session:	Follow-up session	• Address any motivational problems with adolescent and parent
Module 2	Supporting adolescents in developing adaptive skills concerning different situations	• Repetition module 1 and review homework.<br>• Addressing unhelpful avoidance.<br>• Introduction to shared parent-adolescent time.<br>• Introduction to strategies for parents to support their adolescents in facing avoided situations and seeking positive experiences.<br>• **Homework**: Engage in shared parent-adolescent time and supporting adolescents to stay in situations that are avoided or seek out positive situations.
Module 3	Validation	• Repetition module 2 and review homework.<br>• Introduction to the concept of validation.<br>• **Homework**: Engage in shared parent-adolescent time and validation.
Module 4	Supporting adolescents in developing adaptive skills to manage their thoughts	• Repetition module 3 and review homework.<br>• Introduction to common pitfalls when practicing validation.<br>• Introduction to how parents can support their adolescent in changing their attitude toward their thoughts.<br>• **Homework:** Engage in shared parent-adolescent time, validation, and supporting their adolescent with acceptance of thoughts and helpful appraisal styles.
Module 5	Supporting adolescents in developing adaptive skills to manage impulsivity and manage conflicts	• Introduction to conflict management and collaborative problem-solving.<br>**Homework:** Practice collaborative problem-solving.
Module 6		• Summary and maintenance plan

© 2025 Sjöblom K et al. *JAMA Network Open*.

eTable 3. Content of the adolescent program in Supportive Treatment

Module	Name and theme of module	Content
Module 1	Receiving support	• Introduction to mental health.<br>• Reflection on the type and level of support the adolescent needs from oneself and others.<br>• Introduction to fictive characters.<br>• **Homework**: Reflect on their well-being and mental health problems.
Video-link session:	Follow-up session	• Address any motivational problems with adolescent.<br>• Addressing direction for the treatment by reflecting on the "What does improved well-being mean to you?".
Module 2	Self-esteem, self-confidence, and identity	• Repetition module 1 and review homework.<br>• Introduction to self-esteem, self-confidence, and identity.<br>• Psychoeducation about common mental health problems.<br>• **Homework**: Reflect on their self-esteem, and self-confidence during the week and how it can be strengthened.
Module 3	Friendships	• Repetition module 2 and review homework.<br>• Psychoeducation about the reasons for having common mental health problems.<br>• Introduction to and reflection on friendship and how it can affect one's mental health.<br>• **Homework**: Reflect on what defines a good friendship, how they can handle bad relationships and how to find new friends.
Module 4	Family	• Repetition module 3 and review homework.<br>• Psychoeducation about how mental health problems are manifested physically (e.g., headaches, digestive problems, and fatigue).<br>• Introduction to and reflection on family problems and how they might affect one´s mental health.<br>• **Homework:** Reflect on how the family affects one´s mental health and how to make the best of one´s family situation.
Module 5	School	• Repetition module 4 and review homework.<br>• Psychoeducation on reasons for developing mental health issues.<br>• Introduction to and reflection on school and how school might affect one´s mental health.<br>**Homework:** Reflect on what can be done in school to positively impact their mental health and how to manage school stress.
Module 6	Summary	• Repetition module 5 and review homework.<br>• Summary and maintenance plan.

© 2025 Sjöblom K et al. *JAMA Network Open*.

eTable 4. Content of the parent course in Supportive Treatment

**Module**	**Name and theme of module**	**Content**
Module 1	Supporting adolescents in reflecting on receiving support	• Introduction to mental health problems in youth the role of parents in treatment.<br>• Reflection on the types of mental health problems affecting their adolescent.<br>• Introduction to fictive parent characters<br>• **Homework**: Reflect on what could be done to improve the well-being of their adolescent.
Video-link session:	Follow-up session	• Address any motivational problems with adolescent and parent.
Module 2	Supporting adolescents in reflecting on self-esteem, self-confidence, and identity	• Repetition module 1 and review homework.<br>• Introduction to and reflecting on being a parent to an adolescent.<br>• **Homework**: Reflect on the challenges they experience in parenting and on how to strengthen their adolescent's self-esteem.
Module 3	Supporting adolescents in reflecting on friendship	• Repetition module 2 and review homework.<br>• Introduction to and reflecting on their adolescent safety net.<br>• Introduction to and reflection on their adolescent´s internet use.<br>• **Homework**: Reflect on how they can support their adolescent's friendships. Reflect on concerns about their adloescent's internet use.
Module 4	Supporting adolescents in reflecting on family relations	• Repetition module 3 and review homework.<br>• Introduction to and reflecting on their adolescent family relations.<br>• Introduction to and reflection on self-care activities.<br>• **Homework**: Reflect on actions to take to improve family relationships, and self-care activities for themselves.
Module 5	Supporting adolescents in reflecting on school	• Repetition module 4 and review homework.<br>• Introduction to and reflection on how school might affect one´s mental health.<br>**Homework:** The parent is encouraged to reflect on their adolescent school situation and self-care activities for themselves.
Module 6	Summary	• Repetition module 5 and review homework.<br>• Summary and maintenance plan.

© 2025 Sjöblom K et al. *JAMA Network Open*.

eFigure 1. Screenshot of a Work Sheet from Primary Care Online Emotion-regulation Treatment (POET)

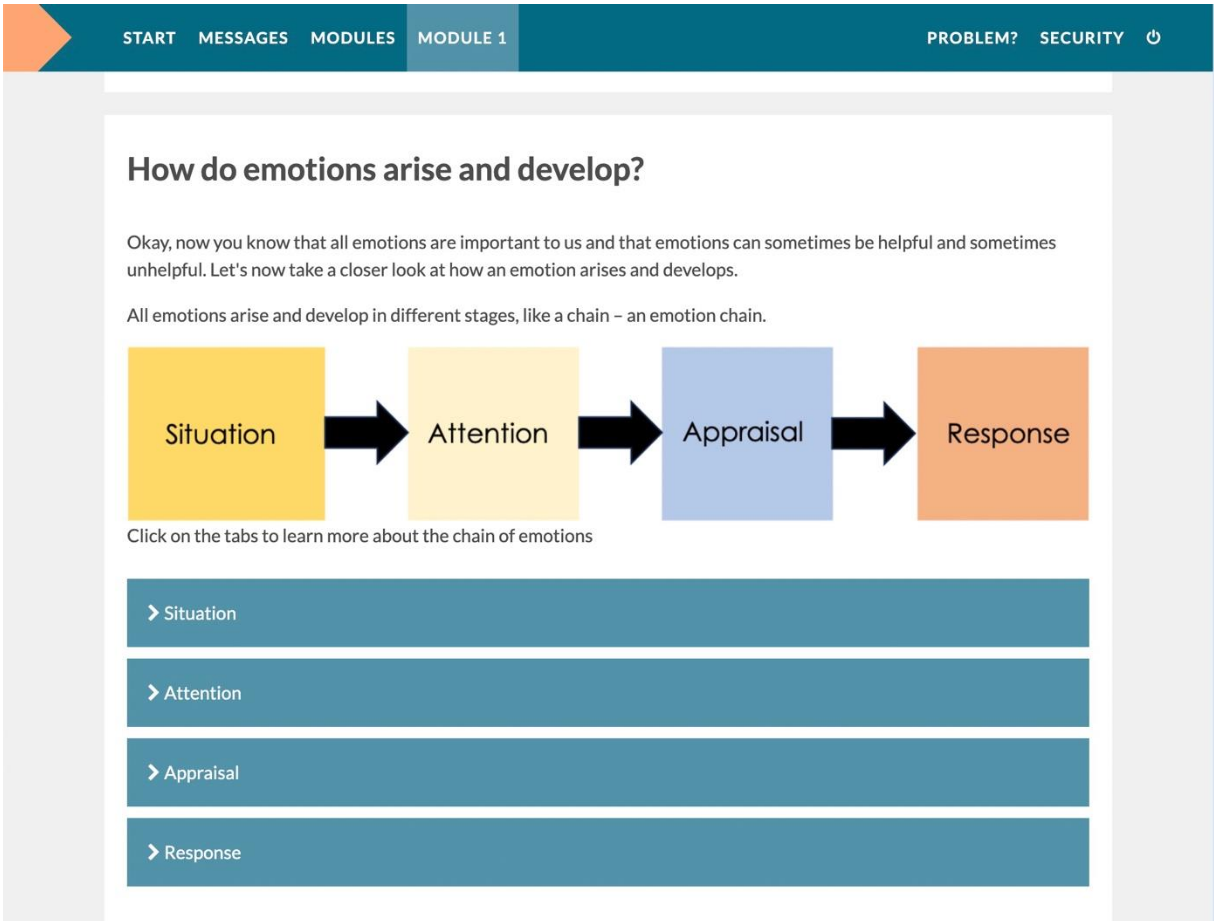

© 2025 Sjöblom K et al. *JAMA Network Open*.

eFigure 2. Screenshot of the message function of both treatments

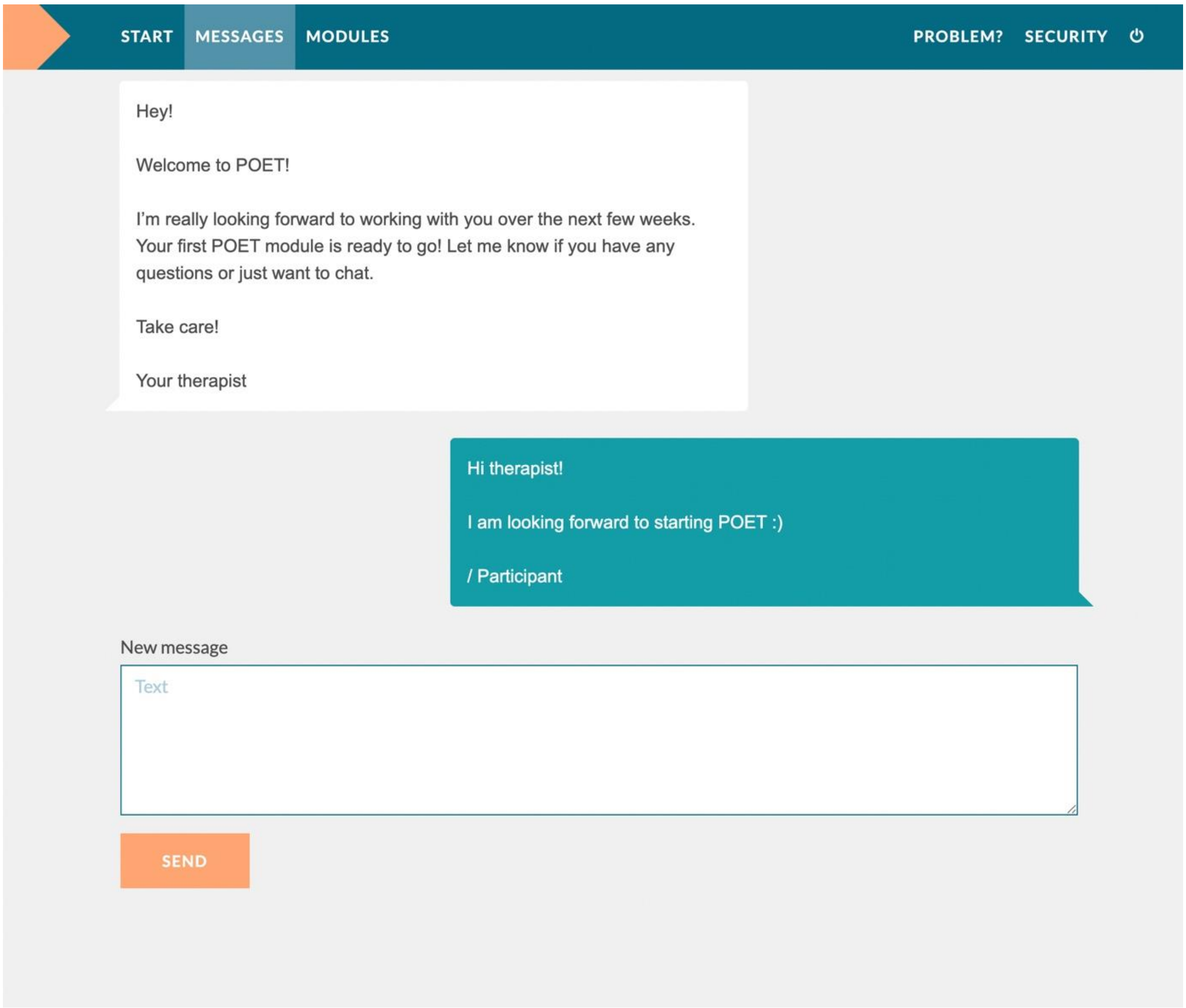

© 2025 Sjöblom K et al. *JAMA Network Open*.

eFigure 3. Screenshot of Work Sheet from the active control treatment (Online Supportive Treatment)

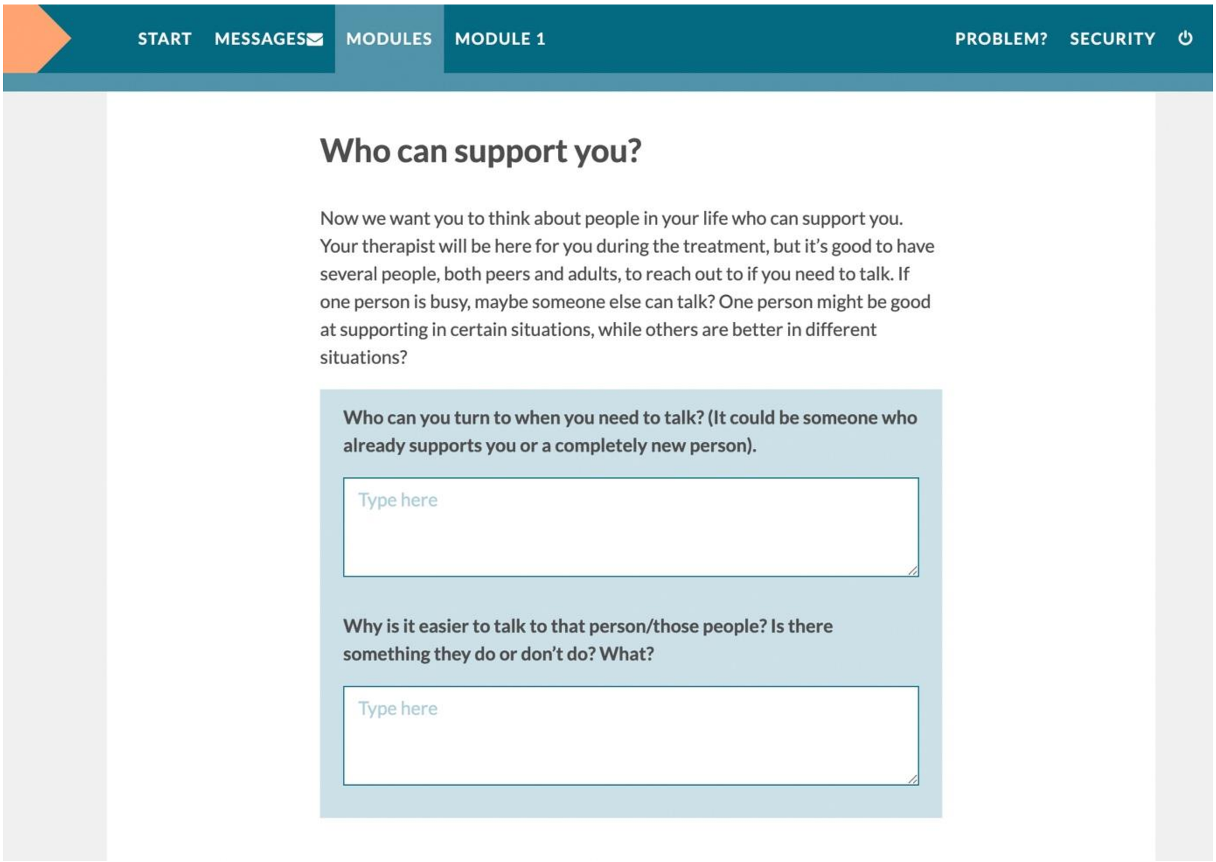

© 2025 Sjöblom K et al. *JAMA Network Open*.

eReferences

1. Sheehan DV, Sheehan KH, Shytle RD, Janavs J, Bannon Y, Rogers JE, et al. Reliability and Validity of the Mini International Neuropsychiatric Interview for Children and Adolescents (MINI-KID). J Clin Psychiatry. 2010 Mar 15;71(03):313–26.

2. Chorpita BF, Yim L, Moffitt C, Umemoto LA, Francis SE. Assessment of symptoms of DSM-IV anxiety and depression in children: a revised child anxiety and depression scale. Behav Res Ther. 2000 Aug 1;38(8):835–55.

3. Busner J, Targum SD. The Clinical Global Impressions Scale. Psychiatry Edgmont. 2007 Jul;4(7):28–37.

4. Shaffer D, Gould MS, Brasic J, Ambrosini P, Fisher P, Bird H, et al. A Children's Global Assessment Scale (CGAS). Arch Gen Psychiatry. 1983 Nov 1;40(11):1228–31.

5. Garnefski N, Kraaij V. Cognitive emotion regulation questionnaire – development of a short 18-item version (CERQ-short). Personal Individ Differ. 2006 Oct 1;41(6):1045–53.

6. Garnefski N, Kraaij V, Spinhoven P. Negative life events, cognitive emotion regulation and emotional problems. Personal Individ Differ. 2001 Jun 1;30(8):1311–27.

7. Preece DA, Mehta A, Petrova K, Sikka P, Bjureberg J, Chen W, et al. The Perth Alexithymia Questionnaire-Short Form (PAQ-S): A 6-item measure of alexithymia. J Affect Disord. 2023 Mar 15;325:493–501.

8. Devilly GJ, Borkovec TD. Psychometric properties of the credibility/expectancy questionnaire. J Behav Ther Exp Psychiatry. 2000 Jun 1;31(2):73–86.

9. Thompson-Hollands J, Bentley KH, Gallagher MW, Boswell JF, Barlow DH. Credibility and outcome expectancy in the unified protocol: Relationship to outcomes. J Exp Psychopathol. 2014;5(1):72–82.

10. Larsen DL, Attkisson CC, Hargreaves WA, Nguyen TD. Assessment of client/patient satisfaction: Development of a general scale. Eval Program Plann. 1979 Jan 1;2(3):197–207.

© 2025 Sjöblom K et al. *JAMA Network Open*.
