# Supplementary material for: Online Transdiagnostic Emotion Regulation Treatment for Adolescents With Mental Health Problems: A Randomized Clinical Trial
Source: JAMA Netw Open. 2025 Jun 11;8(6):e2514871. doi: 10.1001/jamanetworkopen.2025.14871 (PMC12159777; doi:10.1001/jamanetworkopen.2025.14871)
Supplement: Supplement 3. — Data Sharing Statement [file jamanetwopen-e2514871-s003.pdf]

Data Sharing Statement

Sjöblom. Online Transdiagnostic Emotion Regulation Treatment for Adolescents With Mental Health Problems. *JAMA Netw Open*. Published June 11, 2025.
doi:10.1001/jamanetworkopen.2025.14871

Data
Additional Information: ClinicalTrials.gov, https://clinicaltrials.gov/study/NCT05032547, Identifier: NCT05032547
Data available: No

Additional Information
Explanation for why data not available: Patient-level data are not publicly available due to national (Swedish) and EU legislation but could be made available from the corresponding author upon reasonable request following approval from the Swedish Ethical Review Authority.
